# The prevalence of chronic diseases and major disease risk factors at different ages among 150 000 men and women living in Mexico City: cross-sectional analyses of a prospective study

**DOI:** 10.1186/1471-2458-9-9

**Published:** 2009-01-09

**Authors:** Pablo Kuri-Morales, Jonathan Emberson, Jesús Alegre-Díaz, Roberto Tapia-Conyer, Rory Collins, Richard Peto, Gary Whitlock

**Affiliations:** 1 Facultad de Medicina, Universidad Nacional Autónoma de México, Mexico City, Mexico; 2 Clinical Trial Service Unit and Epidemiological Studies Unit (CTSU), University of Oxford, Oxford, UK; 3 Secretaria de Salud, Direccion General de Epidemiologia, Mexico City, Mexico

## Abstract

**Background:**

While most of the global burden from chronic diseases, and especially vascular diseases, is now borne by low and middle-income countries, few large-scale epidemiological studies of chronic diseases in such countries have been performed.

**Methods:**

From 1998–2004, 52 584 men and 106 962 women aged ≥35 years were visited in their homes in Mexico City. Self reported diagnoses of chronic diseases and major disease risk factors were ascertained and physical measurements taken. Age- and sex-specific prevalences and means were analysed.

**Results:**

After about age 50 years, diabetes was extremely common – for example, 23.8% of men and 26.9% of women aged 65–74 reported a diagnosis. By comparison, ischaemic heart disease was reported by 4.8% of men and 3.0% of women aged 65–74, a history of stroke by 2.8% and 2.3%, respectively, and a history of cancer by 1.3% and 2.1%. Cancer history was generally more common among women than men – the excess being largest in middle-age, due to breast and cervical cancer. At older ages, the gap narrowed because of an increasing prevalence of prostate cancer. 51% of men and 25% of women aged 35–54 smoked cigarettes, while 29% of men and 41% of women aged 35–54 were obese (i.e. BMI ≥30 kg/m^2^). The prevalence of treated hypertension or measured blood pressure ≥140/90 mmHg increased about 50% more steeply with age among women than men, to 66% of women and 58% of men aged 65–74. Physical inactivity was highly prevalent but daily alcohol drinking was relatively uncommon.

**Conclusion:**

Diabetes, obesity and tobacco smoking are highly prevalent among adults living in Mexico City. Long-term follow-up of this and other cohorts will establish the relevance of such factors to the major causes of death and disability in Mexico.

## Background

Most of the total global burden from chronic diseases in middle age, and especially from vascular diseases, is now borne by low- and middle-income countries [1]. This is due partly to reductions in the risk of dying young (especially from infectious diseases and nutritional deficiencies) in many of these countries, and probably also to increases in lifestyle characteristics (e.g. tobacco, obesity) known to cause chronic diseases in high-income countries. However, to date, there have been few large epidemiological studies of chronic diseases, or of their causes, in lower income countries. There is consequently a need for a greater understanding of both the distribution of chronic diseases in lower income countries, and the distribution of possible lifestyle risk factors, so that appropriate health care and preventive strategies can be put in place.

Mexico is an example of a large middle-income country in the midst of such a transition, with diabetes, ischaemic heart disease (IHD), stroke and alcoholic liver disease all now leading causes of death in middle-age [2]. In this paper, the age- and sex-specific distributions of a range of chronic diseases and potentially modifiable risk factors is assessed in a reasonably representative sample of over 150 000 adults living in Mexico City (one of the world's most populous cities). The findings are directly relevant to current health care provision and disease prevention in Mexico City, and they may be indirectly relevant to other parts of Latin America and elsewhere.

## Methods

The rationale and methods for the Mexico City Prospective Study have been described previously [3] and are briefly summarised below.

### Recruitment of study population

The study was initiated in the mid-1990s to assess associations of established risk factors, and of possible new risk factors, with the main causes of death in Mexico City. Between April 1998 and September 2004, study teams, each consisting of two or three specially trained nurses, surveyed 159 546 adults aged at least 35 years (52 584 men and 106 962 women) from two urban districts of Mexico City (at an average rate of ~2000 per month). The two districts were selected because they contained a diverse, but settled, mix of long-term residents and relatively recent migrants from throughout Mexico, with people from a wide range of different socio-demographic backgrounds [3]. One district (Coyoacán) has a full spectrum of incomes apart from the extremely high and extremely low; the other (Iztapalapa) has mostly middle and lower incomes, but again without the extremes. During the home visit a baseline questionnaire was completed by means of an interviewer-administered electronic handheld data recorder, physical measurements (including blood pressure and anthropometric measurements) were recorded, and a blood sample was taken [3].

### Baseline questionnaire

Participants were asked whether they had ever been diagnosed with any of the following: diabetes (type unspecified), heart attack, angina, hypertension, stroke, chronic obstructive pulmonary disease (COPD), chronic kidney disease (CKD), liver cirrhosis, peptic ulcer, cancer at several specific sites (lung, prostate, cervix, breast, oesophagus/stomach/intestine, mouth/nose/throat), or any other cancer. Ischaemic heart disease (IHD) was defined as a heart attack or angina.

Participants were asked a range of questions about tobacco, including detailed questions regarding past smoking habits and the number of cigarettes smoked each day. They were also asked about their frequency and intensity of recreational physical activity, their current and previous alcohol drinking habits, and their diet.

### Physical measurements

Height, weight, waist circumference and hip circumference were measured, and body mass index (BMI) was calculated as weight in kilograms divided by the square of height in metres (kg/m^2^). "Obesity" was defined as a BMI of 30 kg/m^2 ^or more. Seated blood pressure was measured on each of three occasions, with the average of the three measurements being used in analyses. "High blood pressure" was defined as treated hypertension, measured systolic blood pressure (SBP) ≥140 mmHg, or measured diastolic blood pressure (DBP) ≥90 mmHg. All physical measurements were entered into handheld data recorders, which automatically queried moderately extreme measurements and prevented highly implausible measurements from being recorded. Further statistical plausibility checks were performed centrally and, if necessary, the values were rechecked by field workers.

### Ethical approval

The study was approved by the Central Oxford Research Ethics Committee and by the National Council on Science and Technology (CONACYT: Consejo Nacional de Ciencia y Tecnología).

### Statistical analyses

The primary analyses of disease and risk factor prevalence were stratified by age and sex. In the tables, participants are shown in five 10-year age groups from 35–44 years to 75–84 years at baseline, excluding the small numbers of participants aged ≥85 years. In the figures, however, information from all men and women is shown, men being separated into five, and women into ten, equally sized age groups (so that each of these groups contains information on just over 10 000 people), with estimates of disease and risk factor prevalence (together with their 95% confidence intervals) being plotted against the mean age of each group. Throughout the Results section, emphasis is placed on reporting estimates rather than testing hypotheses since with a study of this size, even small and clinically unimportant differences can be highly statistically significant. However, where particular significance tests have (for clarity) been reported, the p-values are 2-sided. Analyses were performed using SAS version 8.2 [4] and figures were produced using R version 2.2.1 [5].

## Results

### Prevalence of chronic diseases by age and sex

**Figure 1:**
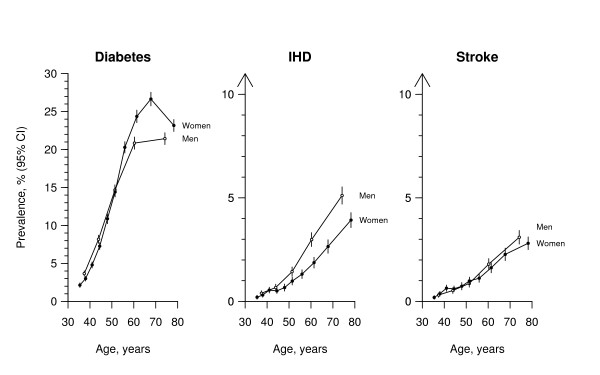
**Sex-specific prevalence of diabetes, IHD and stroke at different ages**. IHD = ischaemic heart disease. Unadjusted prevalences with 95% confidence intervals are shown. Each point involves ~10 000 people.

Table 1 shows the self-reported prevalences of several chronic diseases by age and sex, and Figure 1 displays the prevalences for diabetes, IHD and stroke (together with their associated 95% confidence intervals).

**Table 1:** Age-specific prevalence (%) of some major diseases, by sex (51 768 men and 105 313 women aged 35–84 years)

			**No. (%) with disease**											
			
						**Cancer**								
										
	**Age at resurvey**	**Number recruited**	**Diabetes**	**IHD**	**Stroke**	**Cervix**	**Breast**	**Prostate**	**Other**	**Any**	**COPD**	**CKD**	**Cirrhosis**	**Peptic ulcer**
Men	35–44	16777	804 (4.8%)	72 (0.4%)	61 (0.4%)	-	-	3 (0.0%)	32 (0.2%)	34 (0.2%)	12 (0.1%)	86 (0.5%)	18 (0.1%)	252 (1.5%)

	45–54	13776	1811 (13.1%)	171 (1.2%)	106 (0.8%)	-	-	8 (0.1%)	45 (0.3%)	53 (0.4%)	20 (0.1%)	87 (0.6%)	25 (0.2%)	260 (1.9%)

	55–64	10259	2064 (20.1%)	279 (2.7%)	167 (1.6%)	-	-	25 (0.2%)	58 (0.6%)	81 (0.8%)	52 (0.5%)	122 (1.2%)	48 (0.5%)	255 (2.5%)

	65–74	7300	1736 (23.8%)	352 (4.8%)	202 (2.8%)	-	-	49 (0.7%)	49 (0.7%)	96 (1.3%)	88 (1.2%)	81 (1.1%)	39 (0.5%)	199 (2.7%)

	75–84	3656	712 (19.5%)	198 (5.4%)	127 (3.5%)	-	-	49 (1.3%)	45 (1.2%)	91 (2.5%)	65 (1.8%)	38 (1.0%)	12 (0.3%)	132 (3.6%)

Women	35–44	37591	1474 (3.9%)	146 (0.4%)	166 (0.4%)	170 (0.5%)	57 (0.2%)	-	103 (0.3%)	326 (0.9%)	19 (0.1%)	246 (0.7%)	8 (0.0%)	463 (1.2%)

	45–54	29413	3586 (12.2%)	228 (0.8%)	247 (0.8%)	194 (0.7%)	141 (0.5%)	-	151 (0.5%)	475 (1.6%)	34 (0.1%)	257 (0.9%)	25 (0.1%)	471 (1.6%)

	55–64	19303	4418 (22.9%)	321 (1.7%)	270 (1.4%)	152 (0.8%)	124 (0.6%)	-	126 (0.7%)	396 (2.1%)	51 (0.3%)	232 (1.2%)	30 (0.2%)	340 (1.8%)

	65–74	13025	3505 (26.9%)	392 (3.0%)	299 (2.3%)	116 (0.9%)	70 (0.5%)	-	93 (0.7%)	273 (2.1%)	62 (0.5%)	138 (1.1%)	16 (0.1%)	297 (2.3%)

	75–84	5981	1368 (22.9%)	243 (4.1%)	180 (3.0%)	43 (0.7%)	48 (0.8%)	-	51 (0.9%)	140 (2.3%)	53 (0.9%)	57 (1.0%)	13 (0.2%)	132 (2.2%)

IHD = ischaemic heart disease, COPD = chronic obstructive pulmonary disease, CKD = chronic kidney disease

The standard errors for each of the estimated prevalences above are small: for cancers (< 0.3% [≤ 0.1% excluding age range 75–84 years]); for COPD (≤ 0.2%); for CKD (< 0.2%); for cirrhosis (< 0.1%) and for peptic ulcer (≤ 0.3%). For diabetes, IHD and stroke, 95% confidence intervals around prevalence estimates are shown in Figure 1.

#### Diabetes

23.8% of men and 26.9% of women aged 65 to 74 reported having been diagnosed with diabetes. The prevalence was somewhat lower at younger ages, but even at 35–44 years it was 4.8% in men and 3.9% in women (with standard errors < 0.2%). In early middle age there was a minor excess of males reporting diabetes, whereas at older ages there was a modest excess of females; the prevalence therefore tended to increase slightly more rapidly with baseline age in females than males (Figure 1).

#### IHD and stroke

In each sex, the prevalences of reported IHD and stroke increased continuously with baseline age. At 65–74 years, 4.8% of men and 3.0% of women reported a history of IHD, and 2.8% and 2.3%, respectively, reported a history of stroke (all standard errors < 0.3%). The prevalence of IHD was higher in men than women at all ages, the relative difference being greatest at around 70 years of age, but the absolute difference continuing to widen with age even beyond that (Figure 1). In contrast, the prevalence of prior stroke was similar in men and women up to about age 55, after which there was a slight excess among men.

#### Cancer

At 65–74 years, just 1.3% of men and 2.1% of women reported a history of cancer. At younger ages, a history of cancer was relatively much more common in women than men, due to breast cancer and – especially – to cervical cancer. At older ages the gap narrowed because of an increasing prevalence of prostate cancer. Compared with these three sex-specific cancers, a history of cancer at other specific sites was, at all ages and in each sex, much less common.

#### Other diseases

In each sex the prevalence of COPD at least doubled with each decade of age until old age. The prevalence was always greater in men than women, and the gap widened with age, so by age 65–74, men (1.2%) were more than twice as likely as women (0.5%) to report having the disease (p < 0.00001). In each sex, the prevalence of CKD increased with age up to about 60 years, beyond which it levelled off or possibly decreased. At age 55–84, about 1% of either sex had CKD, but at younger ages, when the prevalences were lower, there was a minor excess of women with this diagnosis. Cirrhosis was generally more prevalent at older than at younger ages, and also among men than among women. For example, about 0.5% of men, compared with about 0.2% of women (p < 0.00001), aged 55–64 reported this condition. A history of peptic ulceration was nearly twice as prevalent at ages 65–74 (men: 2.7%; women 2.3%) as among people three decades younger (men: 1.5%; women 1.2%); both p < 0.00001.

### Prevalence of major disease risk factors, by age and sex

Table 2 shows the prevalences of some behaviours and physical characteristics known to be major disease risk factors in many countries, by age and sex.

**Table 2:** Age-specific prevalences and means of selected risk factors, by sex (51 768 men and 105 313 women aged 35–84 years)

			**Tobacco smoking**						
							
	**Age at resurvey**	**Number recruited**	**Never**	**Former**	**Current**	**Mean (SD) BMI, kg/m^2^**	**Mean (SD) waist:hip ratio**	**Mean (SD) SBP/DBP, mmHg**	**Daily drinking**
Men	35–44	16777	3539 (21.1%)	4313 (25.7%)	8922 (53.2%)	28.0 (4.5)	0.93 (0.06)	123/83 (12/9)	291 (1.7%)

	45–54	13776	2718 (19.7%)	4497 (32.6%)	6559 (47.6%)	28.2 (4.4)	0.95 (0.06)	127/85 (15/10)	333 (2.4%)

	55–64	10259	2034 (19.8%)	4272 (41.7%)	3949 (38.5%)	28.1 (4.2)	0.97 (0.07)	132/86 (17/10)	339 (3.3%)

	65–74	7300	1445 (19.8%)	3685 (50.5%)	2169 (29.7%)	27.6 (4.2)	0.97 (0.07)	136/86 (18/11)	293 (4.0%)

	75–84	3656	845 (23.1%)	2044 (55.9%)	766 (21.0%)	26.7 (3.9)	0.97 (0.07)	136/84 (18/11)	172 (4.7%)

Women	35–44	37591	20855 (55.5%)	6370 (17.0%)	10355 (27.6%)	29.1 (5.2)	0.86 (0.06)	119/79 (13/9)	60 (0.2%)

	45–54	29413	17780 (60.5%)	5210 (17.7%)	6410 (21.8%)	30.1 (5.4)	0.87 (0.07)	126/83 (16/10)	82 (0.3%)

	55–64	19303	13307 (68.9%)	3449 (17.9%)	2544 (13.2%)	30.2 (5.3)	0.89 (0.07)	132/85 (17/10)	75 (0.4%)

	65–74	13025	9484 (72.9%)	2348 (18.0%)	1185 (9.1%)	29.5 (5.2)	0.91 (0.07)	137/85 (18/11)	78 (0.6%)

	75–84	5981	4504 (75.3%)	1141 (19.1%)	334 (5.6%)	28.0 (5.0)	0.92 (0.08)	139/85 (19/11)	58 (1.0%)

Prevalences and means unadjusted. No. with missing values: smoking 48, BMI 1781, blood pressure 314, drinking 62. The median BMI levels for men and women were: 27.6, 27.9, 27.8, 27.3, 26.6; and 28.4, 29.5, 29.7, 29.0, 27.7 respectively

#### Smoking

**Figure 2:**
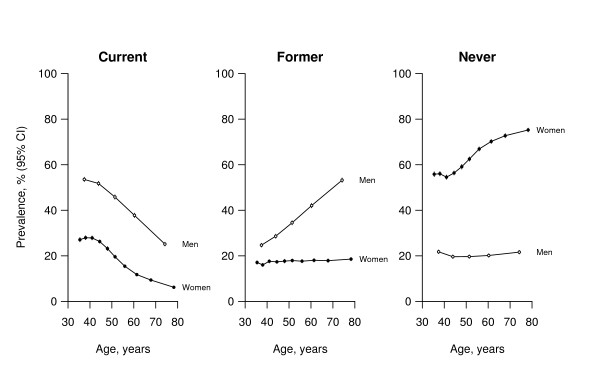
**Sex-specific prevalence of tobacco smoking (current, former and never) at different ages**. Unadjusted prevalences with 95% confidence intervals are shown. Each point involves ~10 000 people.

At all ages, only about 20% of men had never smoked regularly (Figure 2). However, while at age 35–44 there were about twice as many current smokers as ex-smokers, at age 75–84 there were twice as many ex-smokers as current smokers. In contrast, most women had never smoked regularly (56% at age 35–44 increasing to 75% at age 75–84); younger women were much more likely to be current smokers, however, than older women (28% and 6% at ages 35–44 and 75–84 respectively; p < 0.00001). In old age, men were three to four times as likely as women to smoke, but in early middle age they were only twice as likely. Among current smokers, the average number of cigarettes smoked per day was higher among men (about 6–8 cigarettes per day) than women (about 4–6 cigarettes per day; see Additional file 1), and tended to be higher among older people.

#### BMI and the waist to hip ratio

**Figure 3:**
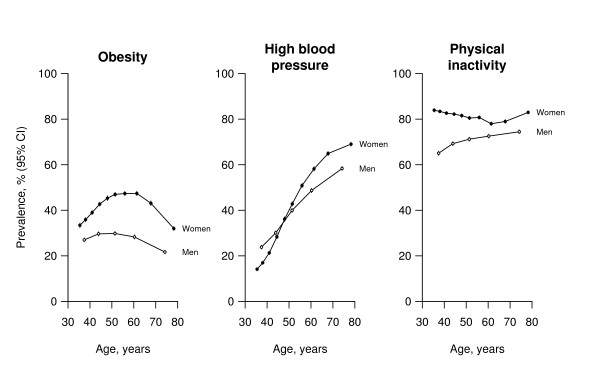
**Sex-specific prevalence of obesity, high blood pressure and physical inactivity at different ages**. Unadjusted prevalences with 95% confidence intervals are shown. Each point involves ~10 000 people. Obesity = BMI ≥ 30 kg/m^2^. High blood pressure = treated hypertension, SBP ≥ 140 mmHg or DBP ≥ 90 mmHg. Physical inactivity = no regular recreational physical activity.

The mean BMI in this population was 27.9 kg/m^2 ^in men and 29.5 kg/m^2 ^in women [3]. In addition to women having higher mean BMI than men, BMI levels were also more widely dispersed in women than men (SD generally 1 kg/m^2 ^larger: Table 2); consequently the prevalence of obesity (BMI ≥ 30 kg/m^2^) was substantially higher in women than men (41% vs 27% respectively; p < 0.00001). In men, obesity was most prevalent between the ages of 40 and 60 (~30%), and least prevalent in old age (though still ~20%); see Figure 3. At all ages, the prevalence was substantially higher in women than men, reaching a maximum of 47% among women between the ages of 50 and 60. The mean waist-to-hip ratio was 0.95 and 0.88 in men and women respectively (p < 0.00001). It increased with age (at least up to age 55–64 in men) and was higher for men than women at all ages.

#### Blood pressure

The mean systolic/diastolic blood pressure (SBP/DBP) was 13/3 mmHg higher at ages 65–74 than at ages 35–44 (i.e., about 30 years younger) for men, and 18/6 mmHg higher for women (the SE's of these differences were ≤ 0.2 mmHg for both SBP and DBP in both sexes). The prevalence of high blood pressure (treated hypertension, SBP ≥140 mmHg or DBP ≥90 mmHg) increased sharply between the ages 35–44 and 75–84, from 26% to 59% in men and, even more sharply from 19% to 70% in women (Figure 3). The difference between men and women in the rate at which SBP increased with age was not much explained by differences in BMI (the F statistic for the interaction term between age and gender was 859 before, and 776 after, adjustment for BMI). Moreover, at least up to about age 65, the mean SBP in women was similar to or lower than that in men despite the mean BMI being 1–2 kg/m^2 ^higher in women.

#### Physical inactivity, alcohol and diet

Recreational physical inactivity was highly prevalent at all ages; overall, 71% of men and 82% of women reported that they did not do any such activity (Figure 3). In both sexes, the prevalence of daily drinking increased steadily with age, but daily drinking was rare even among elderly women (about 1% at 75–84) and not common even among elderly men (about 5% at 75–84). Overall, average fruit and vegetable consumption was marginally higher among women than men (mean [SD] number of days that men and women, on average, ate fruit and vegetables: 4.2 [1.9] vs 4.6 [1.7] days per week; p < 0.00001), and, in both men and women, consumption tended to be a little higher among older people (data not shown). Consumption of fried foods was more common among men than women (mean [SD] number of days that men and women, on average, consumed fried foods: 2.6 [1.9] vs 2.2 [1.7] days per week; p < 0.00001), and was more common among younger people (data not shown).

## Discussion

This study of over 150 000 adults shows the extent and distribution of reported chronic diseases and major disease risk factors prevalent among men and women in Mexico City at the start of the 21^st ^century. Due to the study's size and use of streamlined procedures (including electronically-entered interviewer-administered questionnaires) [3], it has been possible to assess reliably how these reported prevalences vary with age and sex. While the results from this study may not be directly relevant to diseases with a short life expectancy (such as lung cancer: see below), the results are especially relevant to the provision of public health services in Mexico City for chronic conditions such as diabetes, particularly since the study population is reasonably representative of the general population of middle-aged and elderly adults living in this city. Moreover, it is possible that the true disease prevalences are even higher because of the "healthy participant" effect (i.e., the tendency for participants in epidemiological studies to be healthier than the overall population).

In particular, the overall prevalence of diabetes in Mexico City is high, with about one in four adults aged around 70 reporting this condition. This is somewhat higher than has been reported in other surveys in Mexico [6-8], although this may reflect differences in assessment of diabetes (the present study used self-reported medical diagnosis, others have used detailed questionnaires and capillary glucose measurements) or differences in geographical setting (the present study is wholly urban, others have been nationally representative). The higher prevalence among women than men above about age 55 years contrasts with the higher prevalence among men than women at lower ages. This cannot be accounted for by any tendency for one sex to under- (or over-) report diabetes diagnoses, unless that tendency differed qualitatively with age. Nor can it be accounted for by mean BMI level, which was consistently 1–2 kg/m^2 ^higher in women than men at all ages

Diabetes is an important cause of vascular disease, and by about age 70, around 1 in 20 men and 1 in 30 women had a history of IHD (and just over half as many of the men and three-quarters as many of the women had a history of stroke). The higher prevalences among men were despite a lower mean BMI and (at this age) a lower prevalence of diabetes, although the prevalence of smoking – another important cause of vascular disease in many populations – was much higher among men then women. However, the extent to which smoking causes vascular disease in Mexico has not yet been established; the same is true for obesity, diabetes, blood pressure, lipoproteins and possible novel risk factors for vascular disease.

Whereas by about age 70 diabetes was highly prevalent, and a history of vascular disease moderately prevalent, just 1 in 75 men and 1 in 50 women had a history of cancer. However, many cancers kill quite quickly and so would not contribute much to prevalence. For example, in 2005 lung cancer accounted for 1 in 30 male deaths and 1 in 70 female deaths at ages 60–69 in Mexico [9] but it contributed only negligibly to cancer prevalence because of its poor prognosis. The main contributors to cancer prevalence in the present study were three sex-specific cancers that generally have a better prognosis: cancers of the prostate, breast and cervix. Cervical cancer was by far the most prevalent cancer before age 65 in either sex, and cancer at this site is also an unusually common cause of death among middle-aged women in Mexico. For example, in 2005 it was attributed as the cause of death in 24 out of every 100 000 women aged 50–59 years, compared with 5 per 100 000 in the US and 4 per 100 000 in Canada [9]. It is not known whether Mexico's high cervical cancer mortality in middle age is related in some way to its high prevalence of, for example, diabetes.

By around age 70, about 1 in 80 men and 1 in 200 women in this study reported a history of COPD. These prevalences are much lower than the prevalence of significant airway obstruction in a survey of 1000 adults in Mexico City [10], which, although very much smaller than the present study, employed spirometric assessment rather than self-report. There may well be substantial misdiagnosis, including underdiagnosis, of COPD in Mexico as in other Latin American countries [10]. Similarly, there may well be underdiagnosis of CKD [11], so the prevalences in the present report might be substantial underestimates. Furthermore, the possibility of some under-reporting of cirrhosis (and, indeed, of alcohol intake) cannot be excluded: despite the low prevalence in this study (about 1 in 200 men at age 70 and 1 in 800 women), recent national mortality data indicate that some 10% of all male, and 5% of all female, deaths in Mexico at ages 60–69 are attributed to this disease [9]. Studies in other countries indicate that both cirrhosis and CKD can be caused by obesity and by diabetes, but the quantitative relevance of these risk factors to liver or kidney disease in Mexico has not yet been established.

This report demonstrates a high prevalence of tobacco smoking in Mexican men, particularly at younger ages. Half of Mexican men in early middle-age (35–44), and one quarter of Mexican women, smoke tobacco. The observation that the proportion of male current smokers fell with increasing age while the proportion of male ex-smokers rose (Figure 2) is likely to reflect the increased likelihood of quitting with time due, in part, to the accumulation of ill health with increasing age (for instance, the prevalence of IHD was consistently higher among ex-smokers than current smokers for men and women at all ages; Additional file 2).

## Conclusion

Diabetes, obesity and tobacco smoking are highly prevalent among adults living in Mexico City. While the impact of these risk factors on mortality and morbidity in this cohort cannot yet be assessed, continued follow-up will allow detailed estimation of their age- and sex-specific relevance to major causes of death and disability [3]. The unusually high levels of obesity and diabetes in this study may mean that the results will be relevant not only to Mexico, but also to the many countries in which the prevalences of obesity and diabetes are currently increasing.

## Competing interests

The authors declare that they have no competing interests.

## Authors' contributions

PKM, JAD and RTC designed and carried out the study, and contributed to the acquisition of the study's funding. JE and GW drafted the initial manuscript and JE performed the statistical analyses. RC and RP contributed to the design of the study and the acquisition of funding. All authors have contributed to the critical revision of the manuscript for important intellectual content and have approved the final manuscript.

## Pre-publication history

The pre-publication history for this paper can be accessed here:



## Supplementary Material

Additional file 1 **Mean cigarette consumption among current smokers, by age and sex**. Click here for file

Additional file 2 **Prevalence of IHD among current and ex-smokers at different ages**. Unadjusted prevalences with 95% confidence intervals are shown. Click here for file
