# Supplementary figures and images for: The prevalence of chronic diseases and major disease risk factors at different ages among 150 000 men and women living in Mexico City: cross-sectional analyses of a prospective study
Source: BMC Public Health. 2009 Jan 9;9:9. doi: 10.1186/1471-2458-9-9 (PMC2645387; doi:10.1186/1471-2458-9-9)

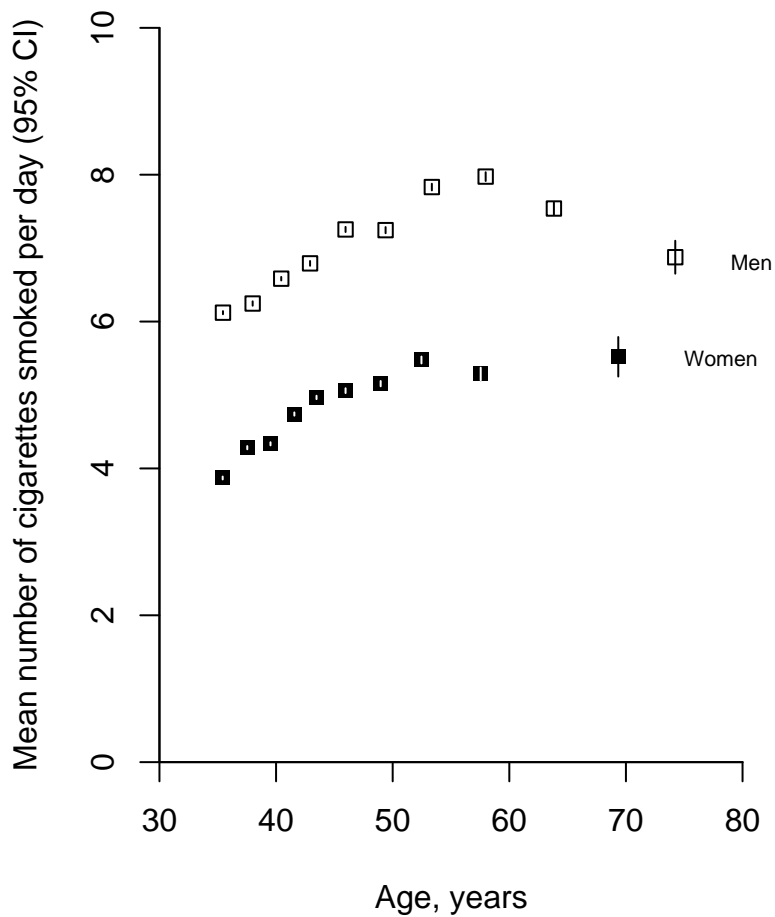

Supplement: Additional file 1 — Mean cigarette consumption among current smokers, by age and sex. [file 1471-2458-9-9-S1.pdf]

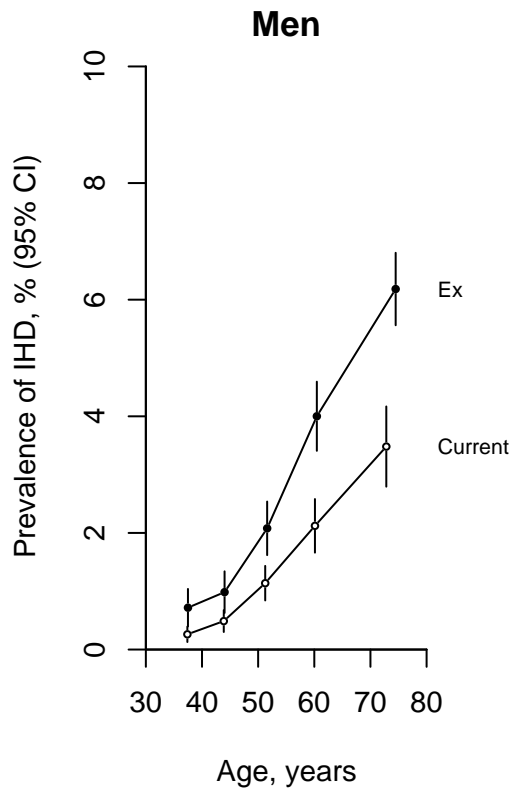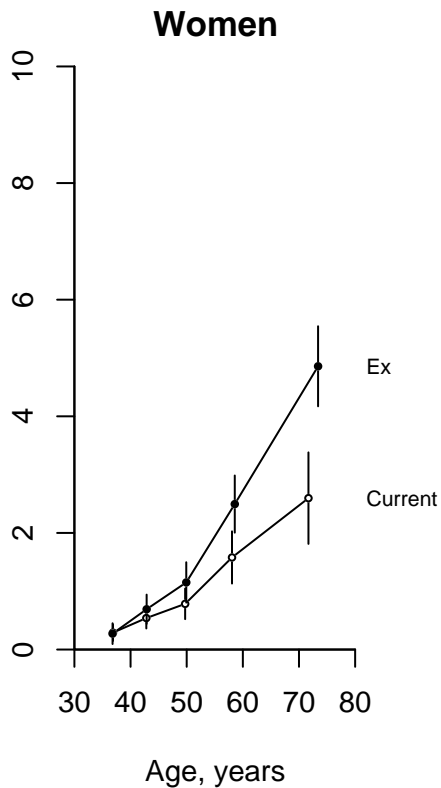

Supplement: Additional file 2 — Prevalence of IHD among current and ex-smokers at different ages. Unadjusted prevalences with 95% confidence intervals are shown. [file 1471-2458-9-9-S2.pdf]
